# Supplementary material for: Precise CAG repeat contraction in a Huntington’s Disease mouse model is enabled by gene editing with SpCas9-NG
Source: Commun Biol. 2021 Jun 23;4:771. doi: 10.1038/s42003-021-02304-w (PMC8222283; doi:10.1038/s42003-021-02304-w)
Supplement: Supplementary file 2 — Supplementary information [file 42003_2021_2304_MOESM2_ESM.pdf]

Supplementary information for

Precise CAG repeat contraction in a Huntington's Disease mouse model is enabled by gene editing with SpCas9-NG

Seiya Oura[1,2,11], Taichi Noda[1,3,11], Naoko Morimura[4], Seiji Hitoshi[4], Hiroshi Nishimasu[5,6], Yoshitaka Nagai[7,8,9], Osamu Nureki[5], Masahito Ikawa[1,2,10,*]

[1]Department of Experimental Genome Research, Research Institute for Microbial Diseases, Osaka University, 3-1 Yamada-oka, Suita, Osaka 565-0871, Japan.

[2]Graduate School of Pharmaceutical Sciences, Osaka University, 1-6 Yamada-oka, Suita, Osaka 565-0871, Japan.

[3]Division of Reproductive Biology, Institute of Resource Development and Analysis, Kumamoto University, 2-2-1 Honjo, Chuo-ku, Kumamoto 860-0811, Japan

[4]Department of Integrative Physiology, Shiga University of Medical Science, Otsu, Shiga, Japan.

[5]Department of Biological Sciences, Graduate School of Science, The University of Tokyo, 7-3-1 Hongo, Bunkyo-ku, Tokyo 113-0033, Japan.

[6]Department of Structural Biology, Research center for Advanced Science and Technology, The University of Tokyo, 4-6-1 Komaba, Meguro-ku, Tokyo 153-8904, Japan.

[7]Department of Degenerative Neurological Diseases, National Institute of Neuroscience, National Center of Neurology and Psychiatry, Kodaira 187-8502, Japan.

[8]Department of Neurotherapeutics, Osaka University Graduate School of Medicine, Osaka 565-0871, Japan.

[9]Department of Neurology, Kindai University Faculty of Medicine, 377-2 Ohnohigashi, Osaka-Sayama, Osaka 589-8511, Japan

[10]Laboratory of Reproductive Systems Biology, Institute of Medical Science, The University of Tokyo, 4-6-1 Shirokanedai, Minato-ku, Tokyo, 108-8639, Japan.

[11]These authors contributed equally: Seiya Oura, Taichi Noda

*To whom correspondence should be addressed. E-mail: ikawa@biken.osaka-u.ac.jp

This PDF file includes:

Supplementary Figure 1 to 10

Other Supplementary materials for this manuscript include the following:

Supplementary Data 1 to 6

Supplementary Movie 1 and 2

Supplementary Figure 1

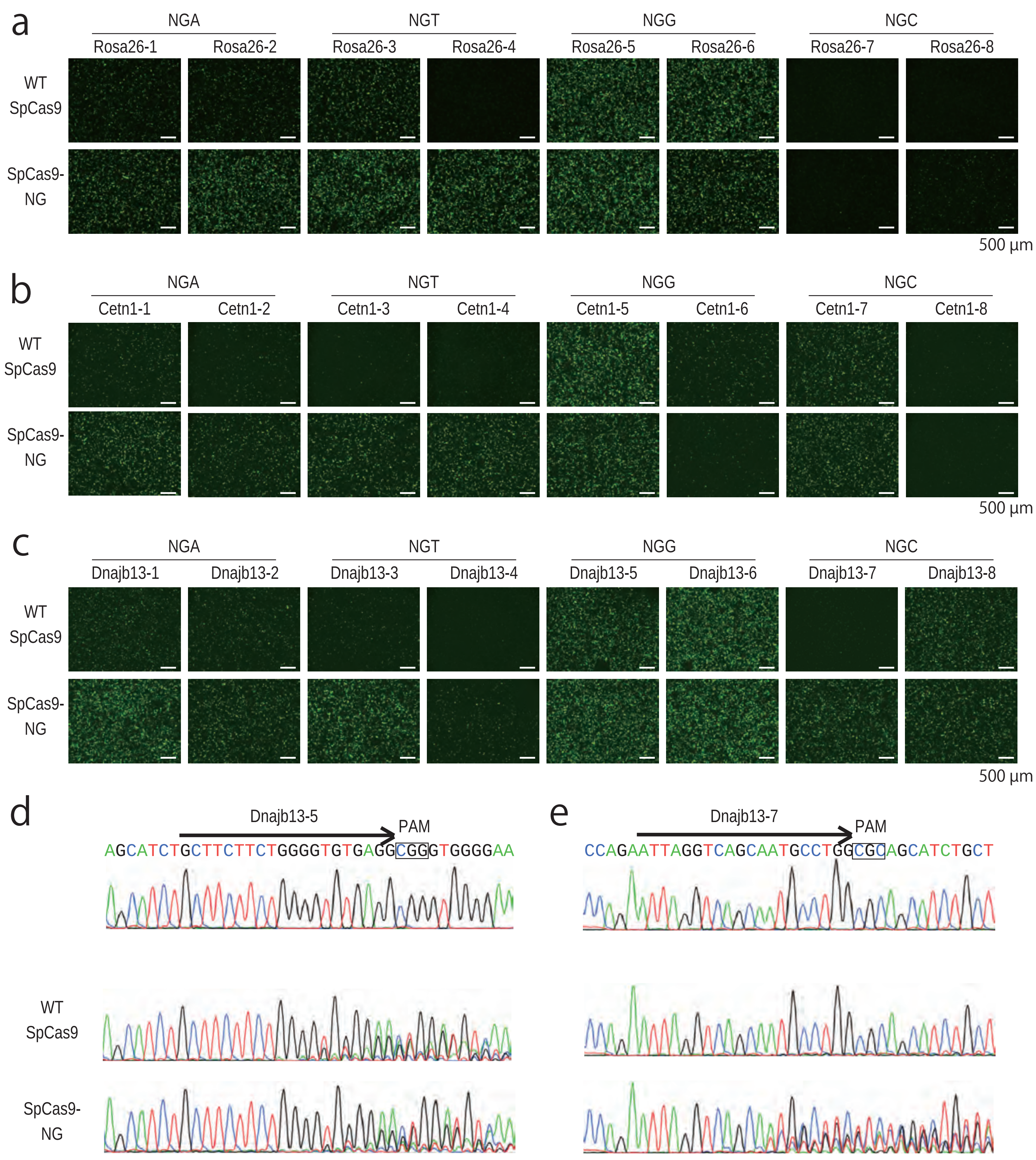

Supplementary Figure 1. Genome editing with SpCas9-NG, related to Fig. 1

a–c The EGFP fluorescence images were taken 36 hours after transfection in the SSA assay.

d, e Sequence of pooled ES cell genome samples. Black arrows and boxes show gRNA target sequence and PAM, respectively.

Supplementary Figure 2

a

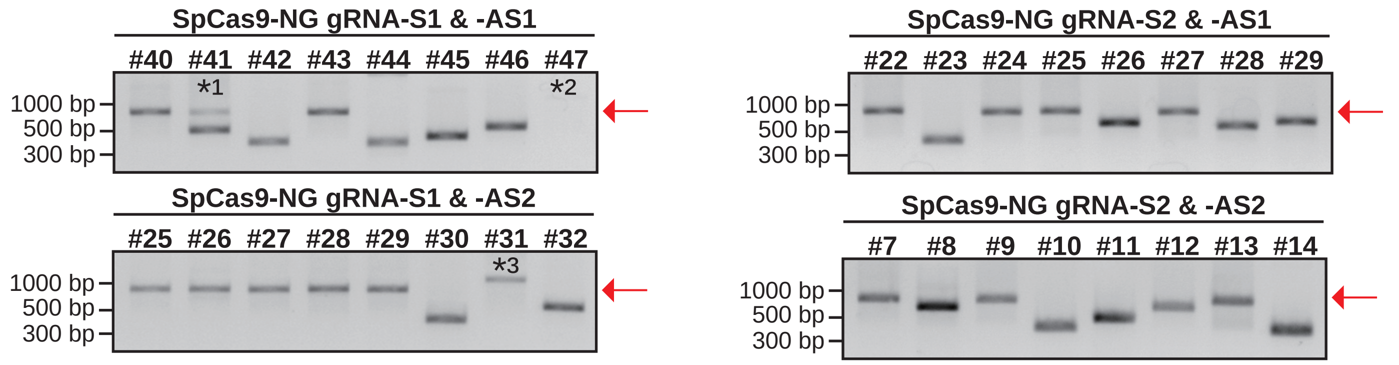

*1 Mosaic *2 No PCR amiplicon *3 Upward-shift

b

gRNA	Examined clones	contracted	in-frame
gRNA S1 & AS1	40	10	2
gRNA S1 & AS2	48	8	3
gRNA S2 & AS1	39	13	4
gRNA S2 & AS2	44	10	1

c

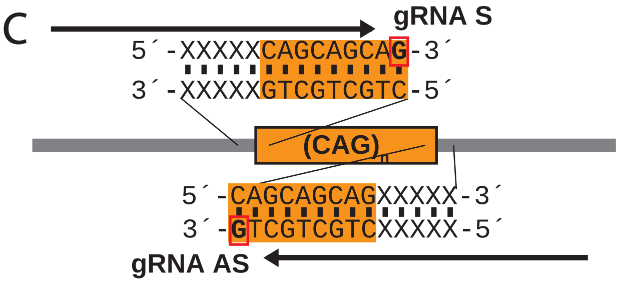

d

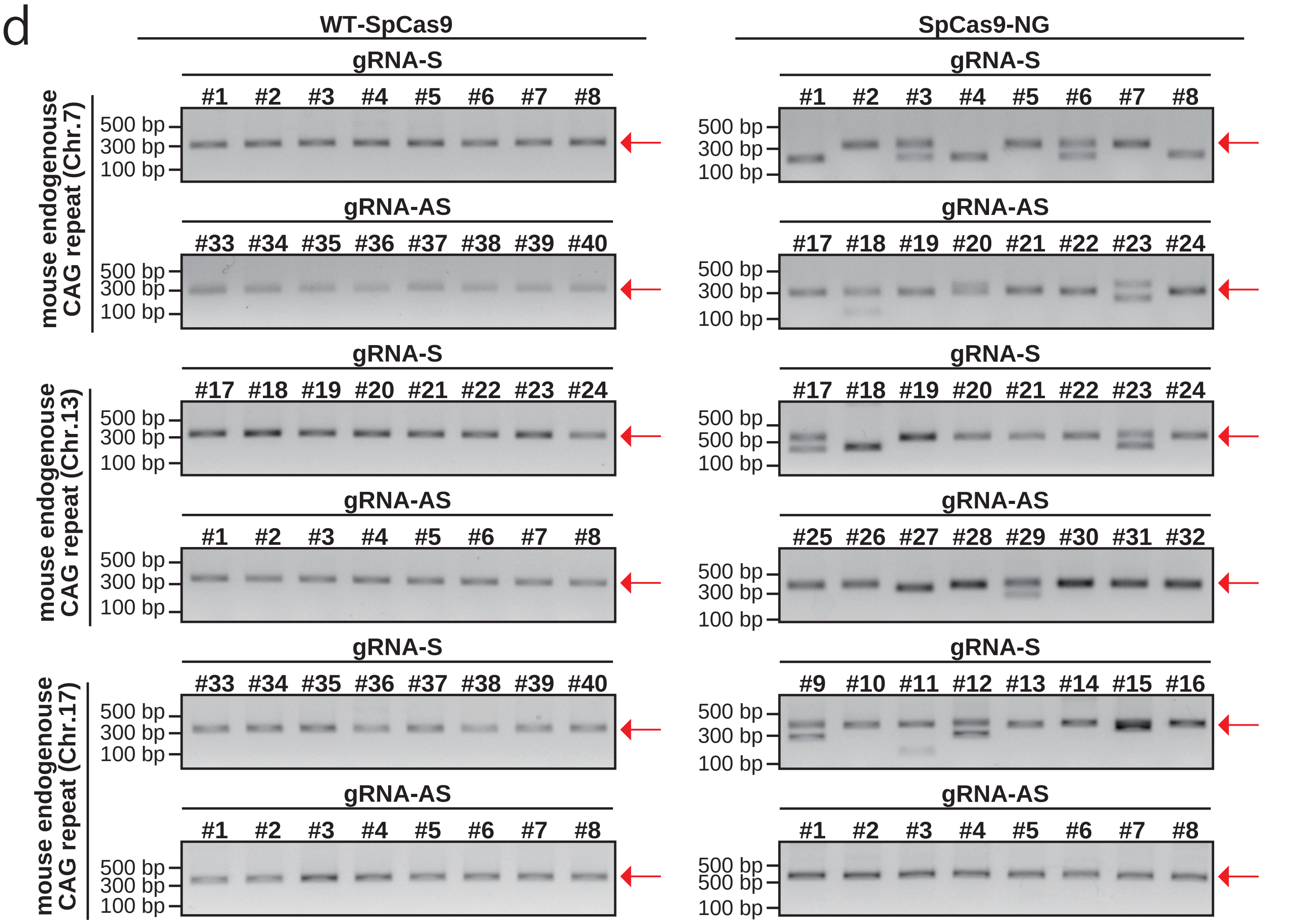

e

Target	gRNA	WT-SpCas9 Clones	WT-SpCas9 Contracted	SpCas9-NG Clones	SpCas9-NG Contracted
CAG repeat (Chr.7)	gRNA S	48	0	43	23
	gRNA AS	48	0	45	2
CAG repeat (Chr.7)	gRNA S	47	0	48	12
	gRNA AS	48	0	43	4
CAG repeat (Chr.7)	gRNA S	48	1	47	18
	gRNA AS	48	0	46	3

Supplementary Figure 2. Two-hit method for CAG repeat contraction and targeting of mouse endogenous CAG repeats, related to Fig. 2

a PCR-based screen for successfully contracted clones. The red arrows indicate the original size of the PCR amplicon. **b** Summary of PCR-based screening in 2A and direct sequencing. **c** Design of gRNAs for targeting mouse endogenous CAG repeats. The orange background color shows the area of the repeat tract. Bold characters with a red box in the DNA sequence indicate the second G in NGN-PAMs. **d** PCR-based confirmation of CAG repeat contraction. The red arrow indicates the original size of the PCR amplicon. **e** Summary of PCR-based confirmation of repeat contraction in 2C.

Supplementary Figure 3

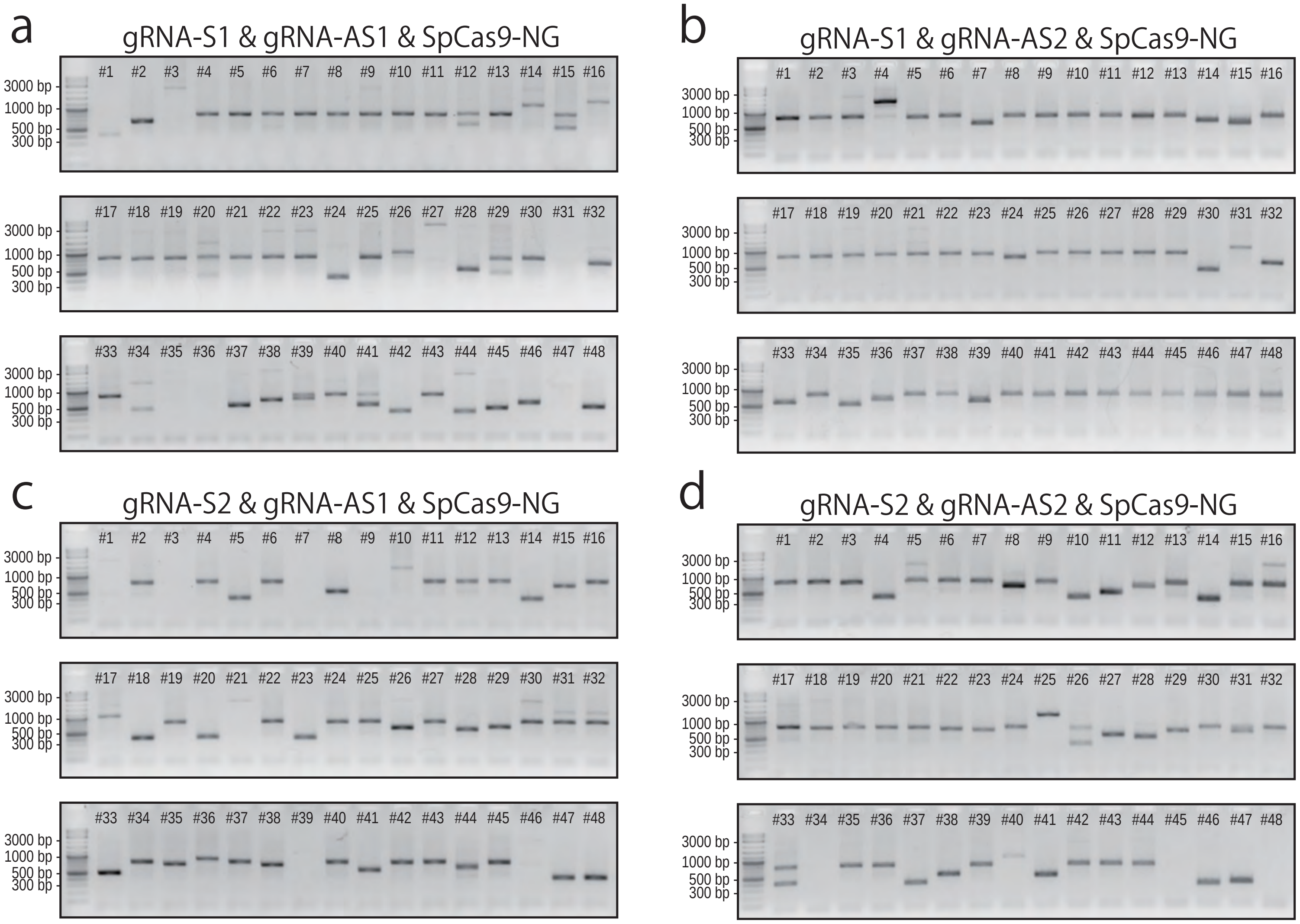

Supplementary Figure 3. Whole gel images of PCR based screeninig of ES cell clones, related to Supplementary Figure 2a.

Supplementary Figure 4

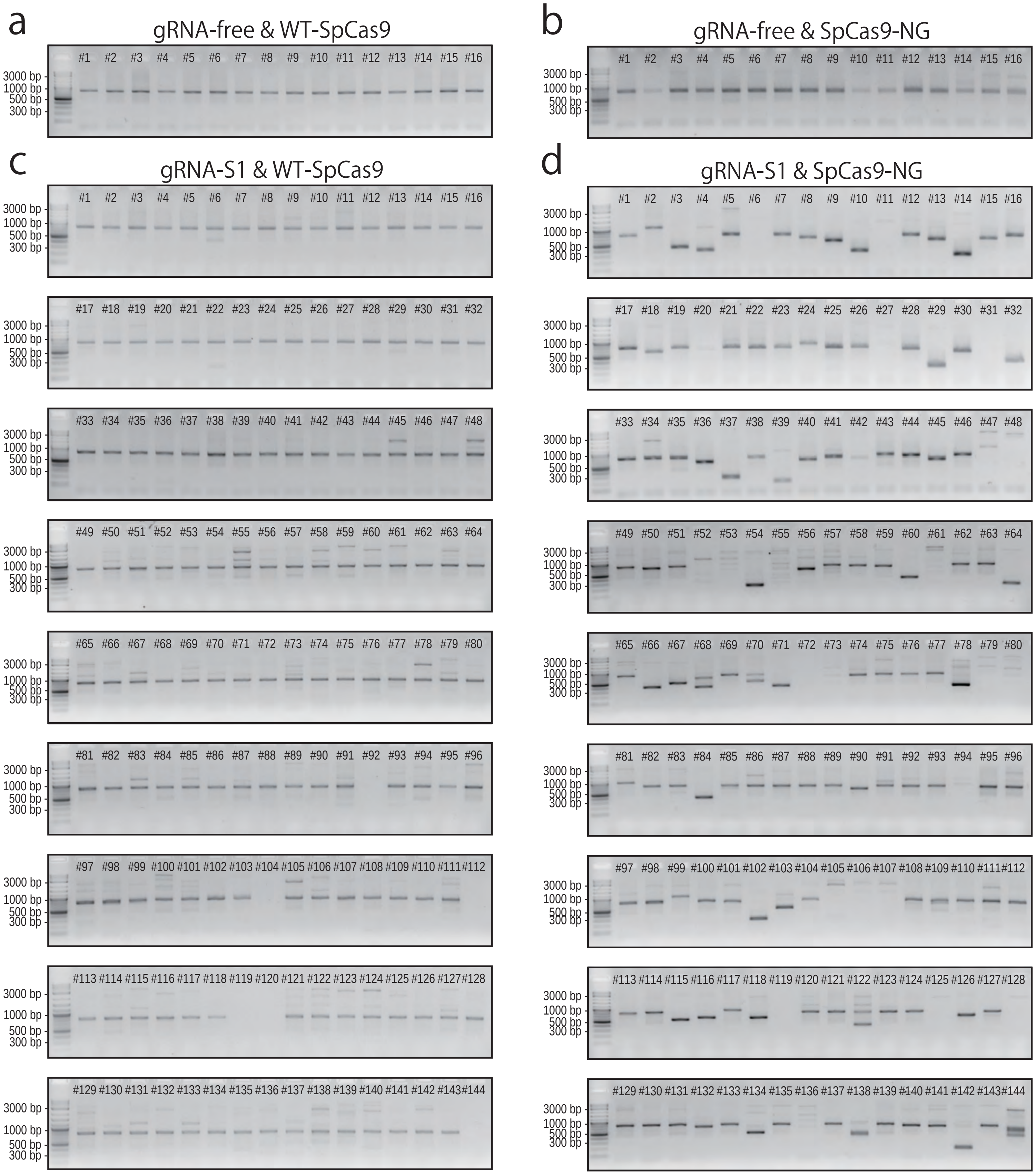

Supplementary Figure 4. Whole gel images of PCR based screeninig of ES cell clones, related to Figure 2c.

Supplementary Figure 5

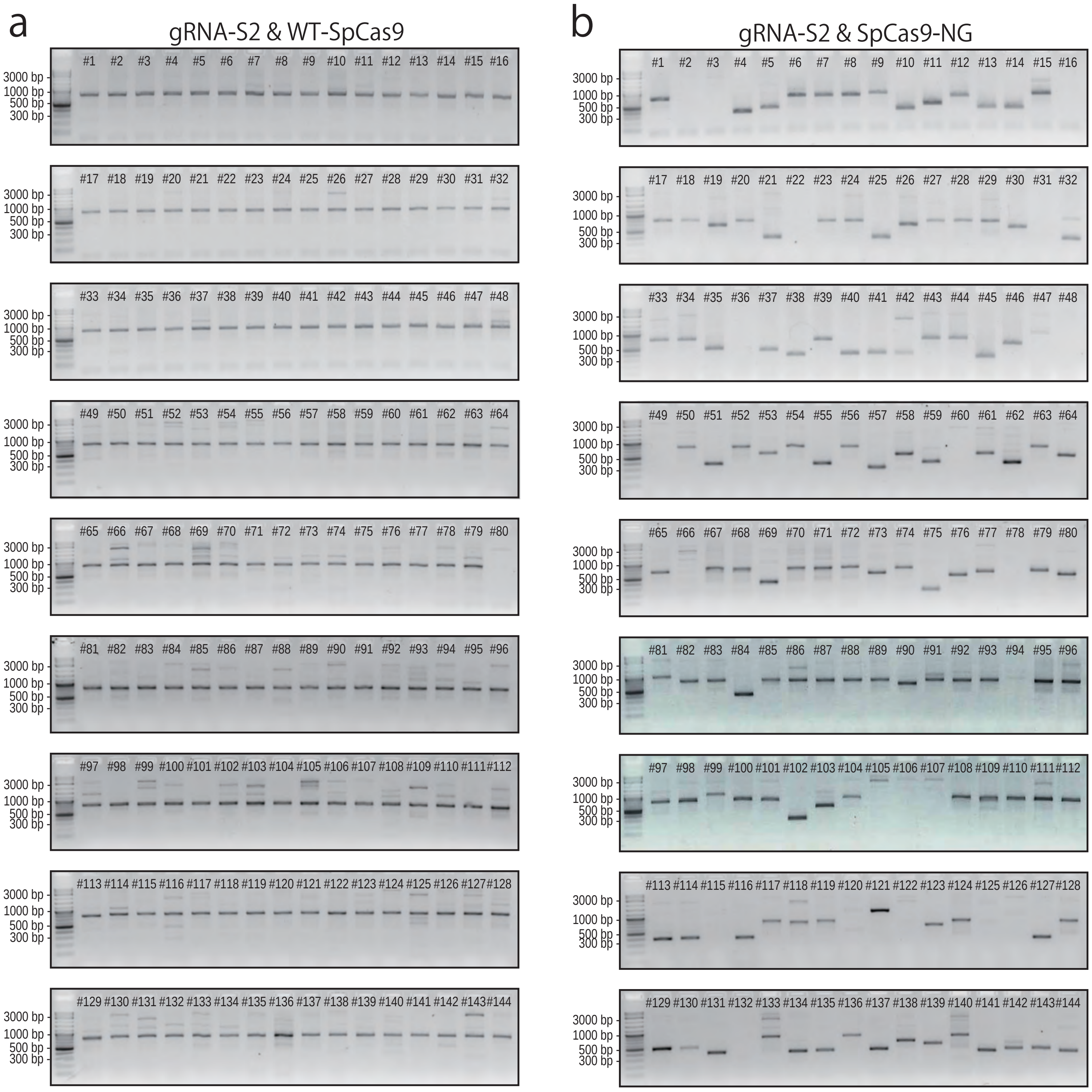

Supplementary Figure 5. Whole gel images of PCR based screeninig of ES cell clones, related to Figure 2c.

Supplementary Figure 6

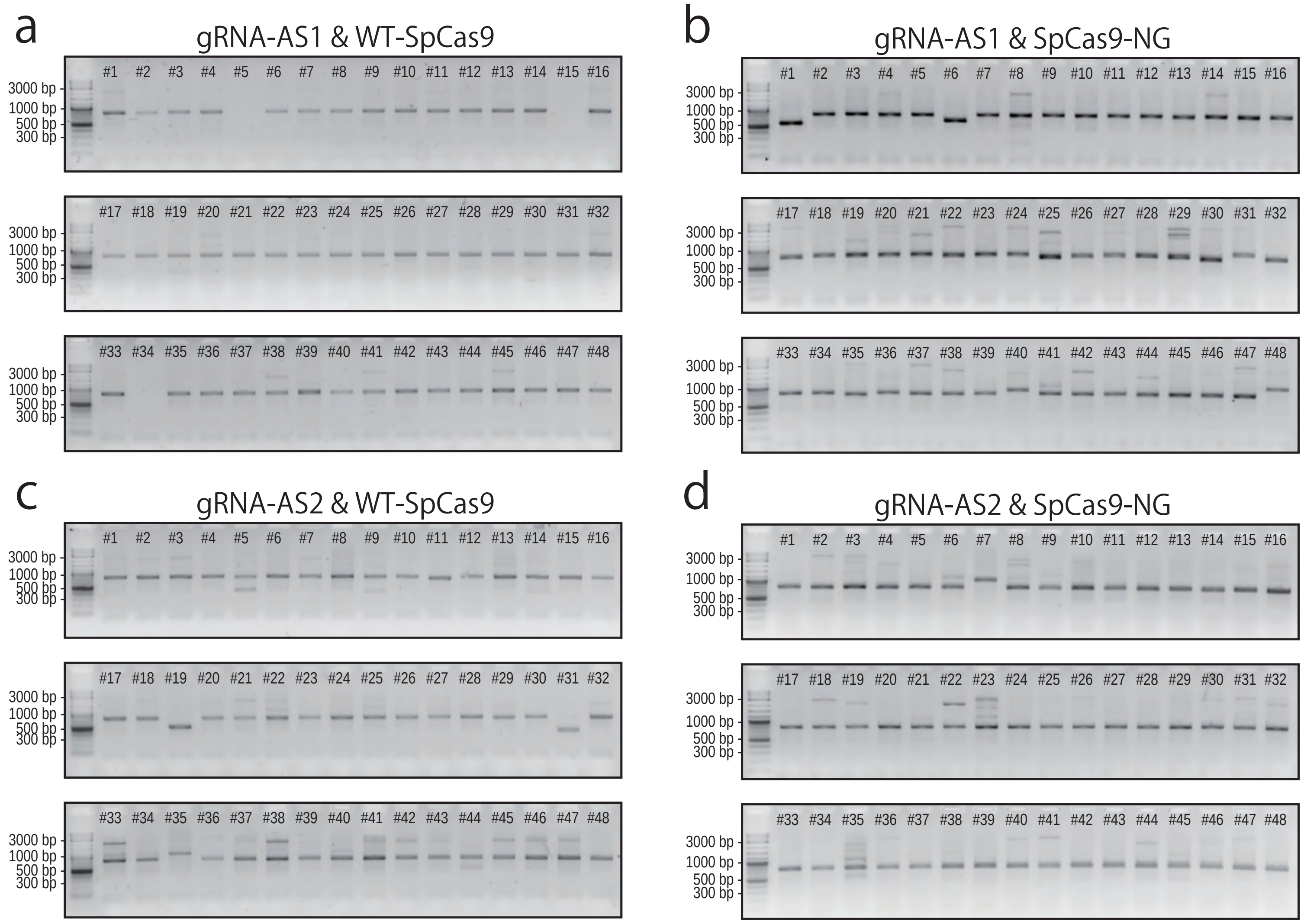

Supplementary Figure 6. Whole gel images of PCR based screeninig of ES cell clones, related to Figure 2c.

Supplementary Figure 7

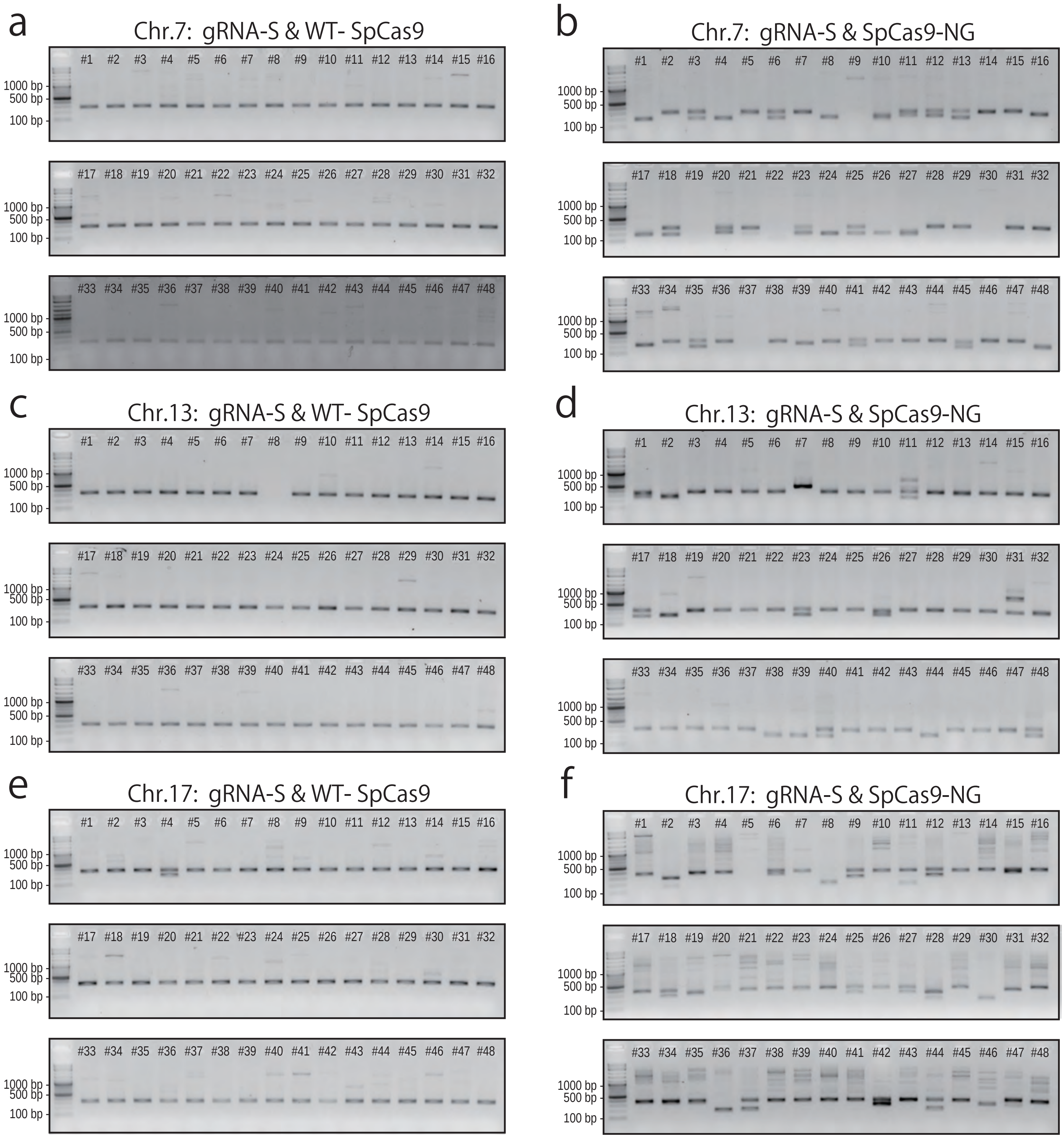

Supplementary Figure 7. Whole gel images of PCR based screeninig of ES cell clones, related to Supplementary Figure 2d.

Supplementary Figure 8

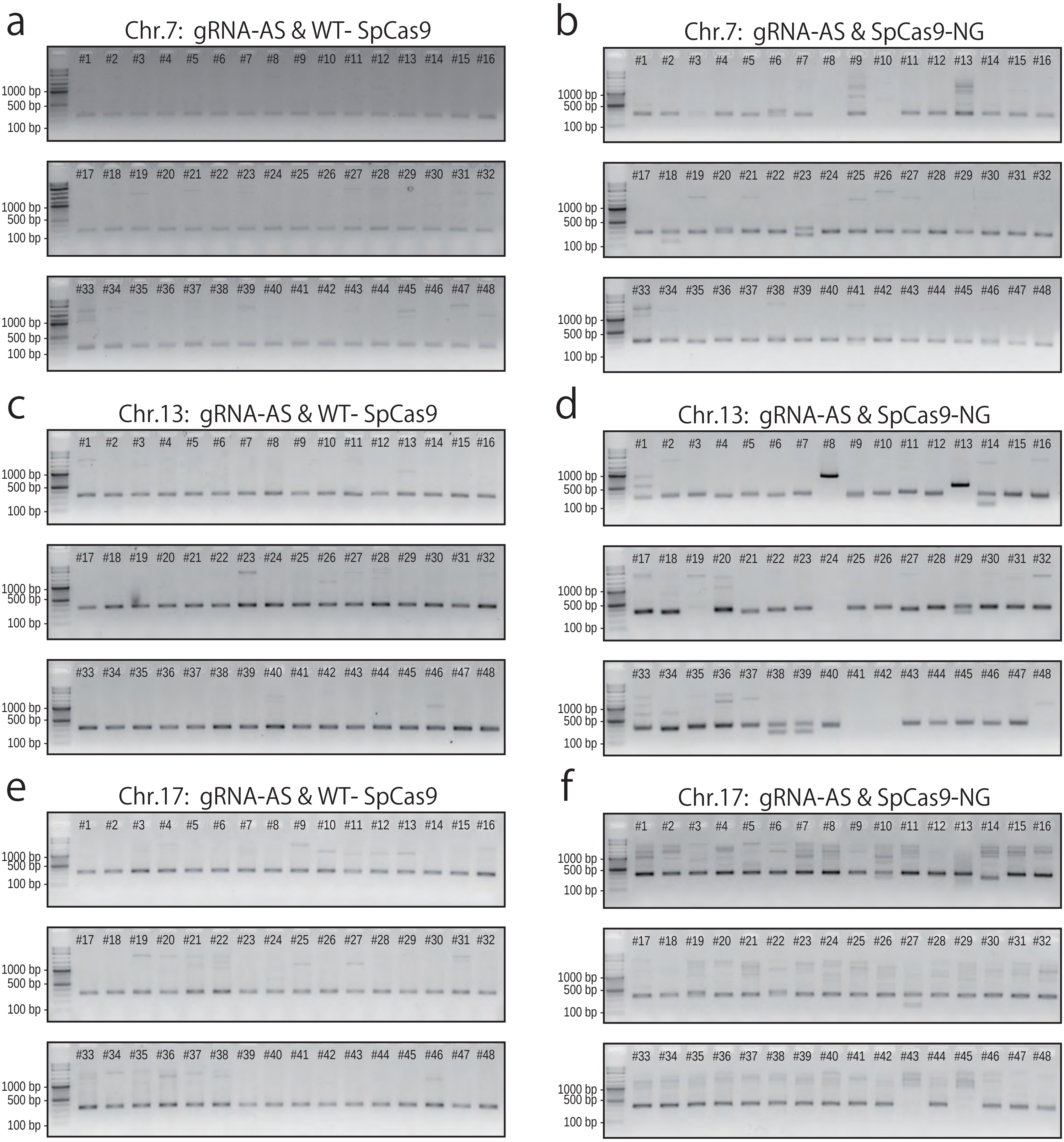

Supplementary Figure 8. Whole gel images of PCR based screeninig of ES cell clones, related to Supplementary Figure 2d.

Supplementary Figure 9

a

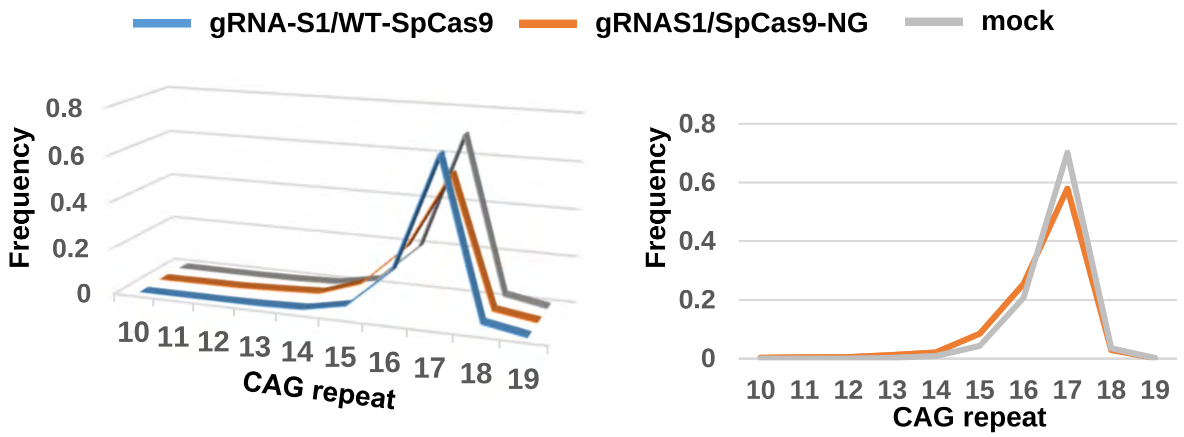

b

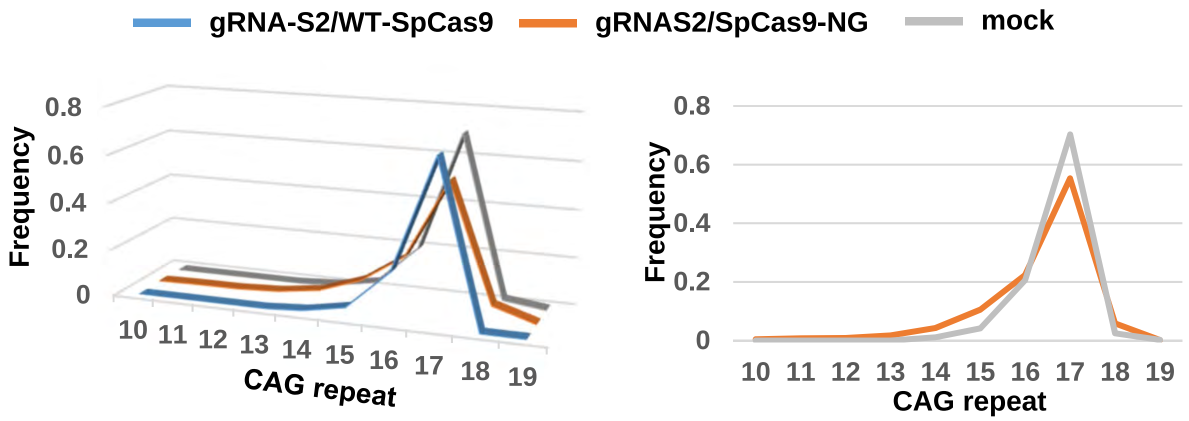

Supplementary Figure 9. Contraction of HTT CAG repeats in HEK293T cells. a–b Histogram of the number of HTT CAG repeat sequences in HEK293T cells transfected with gRNA-S1 (a) and -S2 (b) expression vectors in Figure 3. The frequency of X repeats was calculated by the following formula: [(Reads with X repeats)/ $\sum_{X=10}^{19}$(Reads with X repeats)].

Supplementary Figure 10

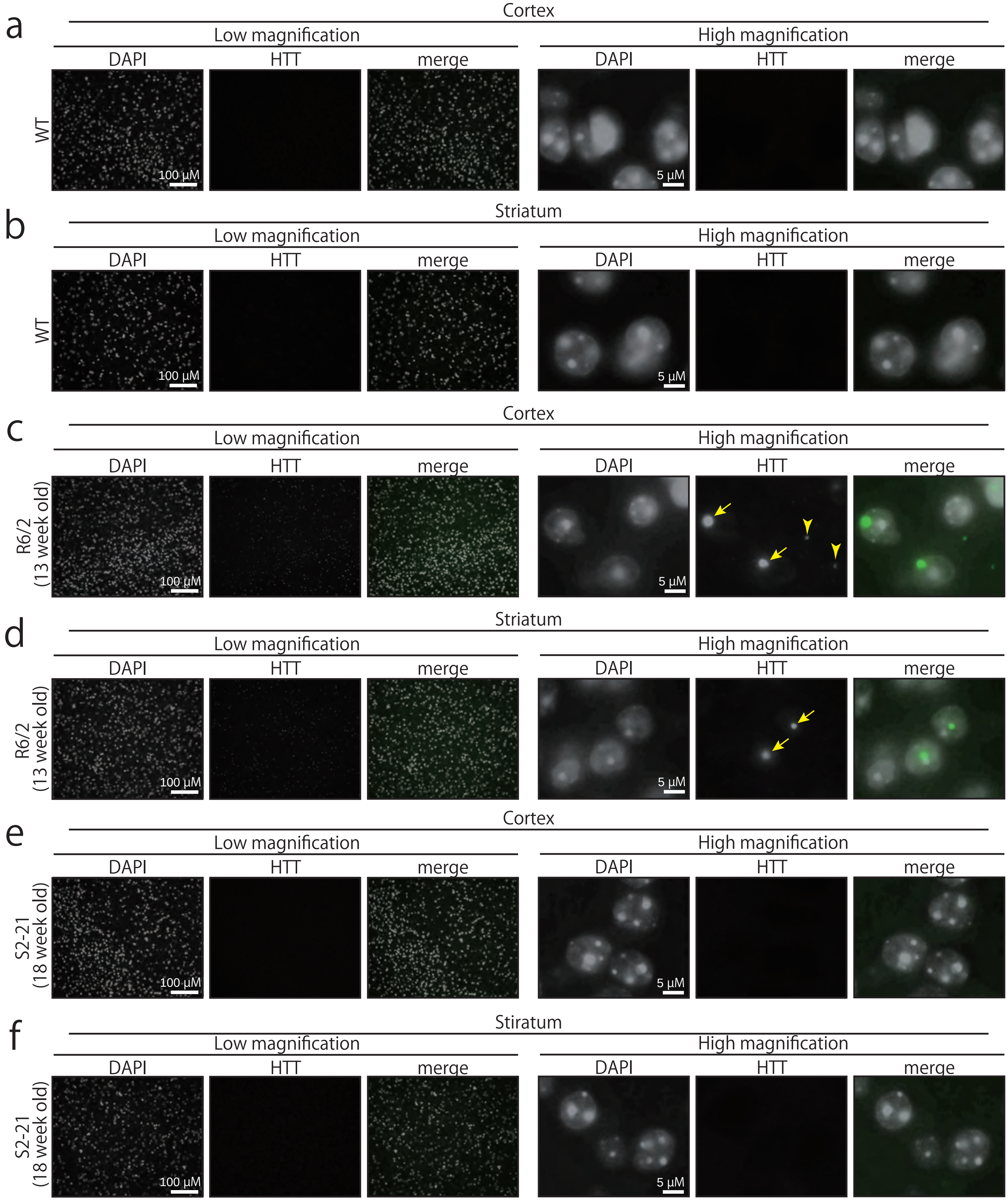

Supplementary Figure 10. HTT aggregation detection by Immunofluorescence, related to Figure 6i–n. a–f Immunofluorescent staining with anti-human HTT antibody. White arrows and arrowheads indicate HTT aggregates inside and outside the nucleus, respectively.sequence and PAM, respectively.
