# Supplementary material for: Precise CAG repeat contraction in a Huntington’s Disease mouse model is enabled by gene editing with SpCas9-NG
Source: Commun Biol. 2021 Jun 23;4:771. doi: 10.1038/s42003-021-02304-w (PMC8222283; doi:10.1038/s42003-021-02304-w)
Supplement: Supplementary file 3 — Description of Supplementary Files [file 42003_2021_2304_MOESM3_ESM.pdf]

Description of Additional Supplementary Files

File name: Supplementary Movie 1

Description: The tail suspension test of R6/2 and genome-edited mice.

File name: Supplementary Movie 2

Description: The behavior of R6/2 and genome-edited mice.

File name: Supplementary Data 1

Description: Primers and gRNAs used in this study.

File name: Supplementary Data 2

Description: Genome editing efficiency in ESCs and zygotes, related to Figure 1d and 1f.

File name: Supplementary Data 3

Description: Sequence of CAG repeat in genome-edited R6/2 ES clones, related to Figure2b-f.

File name: Supplementary Data 4

Description: Bodyweight of chimeric mice, related to Figure 5b and Table1.

File name: Supplementary Data 5

Description: Number of HTT foci in brain sections of chimeric mice, related Figure 5g-h.

File name: Supplementary Data 6

Description: Bodyweight of R6/2 and genome-edited mice, related to Figure 6a-d.
