# Supplementary material for: Precise CAG repeat contraction in a Huntington’s Disease mouse model is enabled by gene editing with SpCas9-NG
Source: Commun Biol. 2021 Jun 23;4:771. doi: 10.1038/s42003-021-02304-w (PMC8222283; doi:10.1038/s42003-021-02304-w)
Supplement: Supplementary file 12 — Reporting Summary [file 42003_2021_2304_MOESM12_ESM.pdf]

nature research

Corresponding author(s): Masahito Ikawa

Last updated by author(s): May 10, 2021

Reporting Summary

Nature Research wishes to improve the reproducibility of the work that we publish. This form provides structure for consistency and transparency in reporting. For further information on Nature Research policies, see our Editorial Policies and the Editorial Policy Checklist.

Statistics

For all statistical analyses, confirm that the following items are present in the figure legend, table legend, main text, or Methods section.

n/a	Confirmed	
☐	☒	The exact sample size (*n*) for each experimental group/condition, given as a discrete number and unit of measurement
☐	☒	A statement on whether measurements were taken from distinct samples or whether the same sample was measured repeatedly
☐	☒	The statistical test(s) used AND whether they are one- or two-sided<br>*Only common tests should be described solely by name; describe more complex techniques in the Methods section.*
☒	☐	A description of all covariates tested
☒	☐	A description of any assumptions or corrections, such as tests of normality and adjustment for multiple comparisons
☐	☒	A full description of the statistical parameters including central tendency (e.g. means) or other basic estimates (e.g. regression coefficient) AND variation (e.g. standard deviation) or associated estimates of uncertainty (e.g. confidence intervals)
☐	☒	For null hypothesis testing, the test statistic (e.g. *F*, *t*, *r*) with confidence intervals, effect sizes, degrees of freedom and *P* value noted<br>*Give P values as exact values whenever suitable.*
☒	☐	For Bayesian analysis, information on the choice of priors and Markov chain Monte Carlo settings
☒	☐	For hierarchical and complex designs, identification of the appropriate level for tests and full reporting of outcomes
☒	☐	Estimates of effect sizes (e.g. Cohen's *d*, Pearson's *r*), indicating how they were calculated

Our web collection on statistics for biologists contains articles on many of the points above.

Software and code

Policy information about availability of computer code

Data collection	Indel efficiency (Fig. 1) was examined using TIDE (https://tide.nki.nl/).
Data analysis	Statistical analyses and plots were generated using R and GraphPad Prism 6 (GraphPad Software). Images were analyzed using ImageJ (NIH) software.

For manuscripts utilizing custom algorithms or software that are central to the research but not yet described in published literature, software must be made available to editors and reviewers. We strongly encourage code deposition in a community repository (e.g. GitHub). See the Nature Research guidelines for submitting code & software for further information.

Data

Policy information about availability of data

All manuscripts must include a data availability statement. This statement should provide the following information, where applicable:

- Accession codes, unique identifiers, or web links for publicly available datasets
- A list of figures that have associated raw data
- A description of any restrictions on data availability

The authors declare that the data that support the findings of this study are available from the corresponding author upon request.

Field-specific reporting

Life sciences study design

All studies must disclose on these points even when the disclosure is negative.

Sample size	Sample sizes were determined based on published studies in the field or our previous experiences. No statistics was used to predetermine the sample size.
Data exclusions	No samples or animals were excluded. Also, the criteria were not pre-established in experiments.
Replication	All experiments were performed using at least three biological replicas.
Randomization	Samples are defined by their unique genotypes. Therefore, no sample randomization was performed.
Blinding	The investigators were not blinded for group allocation.

Reporting for specific materials, systems and methods

We require information from authors about some types of materials, experimental systems and methods used in many studies. Here, indicate whether each material, system or method listed is relevant to your study. If you are not sure if a list item applies to your research, read the appropriate section before selecting a response.

Materials & experimental systems

n/a	Involved in the study	
☐	☒	Antibodies
☐	☒	Eukaryotic cell lines
☒	☐	Palaeontology and archaeology
☐	☒	Animals and other organisms
☒	☐	Human research participants
☒	☐	Clinical data
☒	☐	Dual use research of concern

Methods

n/a	Involved in the study	
☒	☐	ChIP-seq
☒	☐	Flow cytometry
☒	☐	MRI-based neuroimaging

Antibodies

Antibodies used	anti-OCT3/4 antibody (PM048, MBL)<br>anti-huntingtin antibody (MAB5374, Merck)<br>anti-β III tubulin mouse antibody (T8660, Merck)
Validation	All antibodies were validated by the manufacturer or by previously published studies to be suitable for immunofluoresence.

Eukaryotic cell lines

Policy information about cell lines

Cell line source(s)	HEK293T cell line was gifted from Dr. Verma Lab (The Salk Institute).<br>EGR-G101 embryonic stem cell line was generated in the Dr. Ikawa Lab (RIKEN-BRC: AES0182).<br>R6/2-embryonic stem cell were established in this study (will be deposited).
Authentication	Cell lines were authenticated based on their morphology and growth.
Mycoplasma contamination	Mycoplasma contamination was examined using TaKaRa PCR Mycoplasma Detection Set (Takara; 6601).<br>HEK293 and EGR-G101 were tested and nagative for mycoplasma contamination. R6/2 were have not been tested yet.
Commonly misidentified lines (See ICLAC register)	No commonly misidentified lines were used.

Animals and other organisms

Policy information about studies involving animals; ARRIVE guidelines recommended for reporting animal research

Laboratory animals	B6D2F1 (C57BL/6 × DBA2) female mice (6-8 weeks old) used for embryo donor, ICR female mice (indeterminate age) used for either surrogate or foster mother and C57BL/6 used for mating were purchased from Japan SLC.
Wild animals	This study did not involve wild animals.

Field-collected samples	This study did not involve field-collected samples.
Ethics oversight	All animal experiments were approved by the Animal Care and Use committee of the Research Institute for Microbial Diseases, Osaka University (AP 30-01-0).

Note that full information on the approval of the study protocol must also be provided in the manuscript.
